# STING expression and response to treatment with STING ligands in premalignant and malignant disease

**DOI:** 10.1371/journal.pone.0187532

**Published:** 2017-11-14

**Authors:** Jason R. Baird, Zipeng Feng, Hong D. Xiao, David Friedman, Ben Cottam, Bernard A. Fox, Gwen Kramer, Rom S. Leidner, R. Bryan Bell, Kristina H. Young, Marka R. Crittenden, Michael J. Gough

**Affiliations:** 1 Earle A. Chiles Research Institute, Robert W. Franz Cancer Center, Providence Portland Medical Center, Portland, OR, United States of America; 2 Oregon Health and Sciences University, Portland, OR, United States of America; 3 Pathology, Providence Portland Medical Center, Portland, OR, United States of America; 4 The Head and Neck Surgical Institute, Portland, OR, United States of America; 5 The Oregon Clinic, Portland, OR, United States of America; Georgetown University, UNITED STATES

## Abstract

Human papilloma virus positive (HPV^+^) tumors represent a large proportion of anal, vulvar, vaginal, cervical and head and neck squamous carcinomas (HNSCC) and late stage invasive disease is thought to originate from a premalignant state. Cyclic dinucleotides that activate STimulator of INterferon Genes (STING) have been shown to cause rapid regression of a range of advanced tumors. We aimed to investigate STING ligands as a novel treatment for papilloma. We tested therapies in a spontaneous mouse model of papilloma of the face and anogenital region that histologically resembles human HPV-associated papilloma. We demonstrate that STING ligands cause rapid regression of papilloma, associated with T cell infiltration, and are significantly more effective than Imiquimod, a current immunotherapy for papilloma. In humans, we show that STING is expressed in the basal layer of normal skin and lost during keratinocyte differentiation. We found STING was expressed in all HPV-associated cervical and anal dysplasia and was strongly expressed in the cancer cells of HPV^+^ HNSCC but not in HPV-unrelated HNSCC. We found no strong association between STING expression and progressive disease in non-HPV oral dysplasia and oral pre-malignancies that are not HPV-related. These data demonstrate that STING is expressed in basal cells of the skin and is retained in HPV^+^ pre-malignancies and advanced cancers, but not in HPV-unrelated HNSCC. However, using a murine HNSCC model that does not express STING, we demonstrate that STING ligands are an effective therapy regardless of expression of STING by the cancer cells.

## Introduction

As our ability to detect cancerous cells in patients improves, there is an opportunity to treat early tumors before they become invasive and metastatic. Most cancers are known to exhibit a premalignant state, where an expanded population of abnormal cells disrupts normal tissue organization. This premalignant state can exist for years and may remain, resolve, or progress to invasive cancer. Cancer screening programs—for example those based around the Papanicolaou (Pap) smear for cervical carcinoma—aim to identify these abnormal cells for intervention before further malignant transformation. Vaccination against HPV can prevent HPV infection and thus, in principle, prevent HPV-associated malignancies. However, once tumors develop, vaccination against the virus has limited potential impact on progression, perhaps because prophylactic vaccination is directed to neutralizing antibody response to free virions rather than effector T cell control of infected cells [1]. Current therapies for an abnormal Pap smear include surgical excision or ablation. Immunotherapies, including Interferon alpha and Imiquimod have been added to excisional therapies to decrease the rate of recurrence; however, in randomized clinical trials it was found that neither approach impacts the rate of recurrence of cervical dysplasia [2, 3]. In view of the limitations in current therapies, novel treatment options are needed.

In the transgenic pancreatic ductal adenocarcinoma mouse model, Cre expression under a pancreas-specific promoter activates expression of the mutant tumor-driving genes *Kras*^(G12D)^ and *Tp53*^(R172H)^, leading to progressive carcinogenesis from pancreatic intraepithelial neoplasia [4]. In addition, due to leaky Cre expression at variable penetrance, these mice spontaneously develop papillomas of the face and vulva that closely resembles HPV-associated papilloma in humans [4, 5]. The development of these papillomas provides an opportunity to apply this model for investigation of novel treatment approaches to premalignant disease in immunocompetent mice.

Recently, we and others have demonstrated that cyclic dinucleotide (CDN) ligand activation of the STimulator of INterferon Genes (STING) pathway strongly induces type I IFN and TNFα, resulting in rapid regression of a range of advanced tumors [6, 7]. We therefore tested the effect of STING ligands on papilloma in mice. Following direct injection, we observed rapid regression of papilloma following short courses of treatment. To evaluate potential translation to premalignant disease in humans, we evaluated STING expression by immunohistology in clinical tissue specimens. We found that STING is expressed in basal cells of the skin and is retained in HPV^-^associated premalignancies and advanced cancers, but not in HPV^-^ HNSCC derived from non-basal cells. Since treatment with STING ligands causes rapid regression of spontaneous murine papilloma this may represent an advance in the treatment of virus-associated and premalignant diseases. However, STING expression by malignant cells is not essential for treatment of advanced cancers.

## Materials and methods

### Ethics

All animal protocols were approved by the Earle A. Chiles Research Institute IACUC (Animal Welfare Assurance No. A3913-01). De-identified human tissue sections were obtained under IRB# 12–075 approved by the Providence Portland Medical Center IRB.

### Animal models

Pdx-Cre^+/-^ (Stock#014647, Jackson Laboratories, Bar Harbor, ME), Kras^(G12D)+/-^ (Stock#008179, Jackson Laboratories), and Trp53^(R172H)+/-^ (Stock#01XM2, NCI Fredrick Mouse Repository) mice were crossed to generate Pdx-Cre^+/-^ Kras^(G12D)+/-^ Trp53^(R172H)+/-^that generate pancreatic tumors [4]. At variable penetrance, mice develop papilloma of the face and anogenital region [4, 5]. Mice bearing papilloma with no evidence of pancreatic tumors and less than 90d of age were accrued to this study. Masses were treated by injection of 25μg of the cyclic dinucleotide (CDN) c-di-GMP (Invivogen, San Diego, CA), Imiquimod (Invivogen) or PBS vehicle, using a Hamilton syringe for small volume injections. In appropriate experiments, CD8 T cells were depleted by i.p. injection of 50μg anti-CD8 antibody (YTS 169.4 –BioXCell, West Lebanon, NH) one day before treatment and again 1 week later. Cell depletion was confirmed by quantitative flow cytometry of whole blood using fluorescent-conjugated antibodies to CD3 and CD8 purchased from Ebioscience (San Diego, CA) and quantified using AccuCheck fluorescent beads (Invitrogen, Carlsbad, CA) with samples analyzed on a BD LSRII flow cytometer as previously described [8]. The SCCVII squamous cell carcinoma cell line was kindly provided in 2014 by Dr. Lee (Duke University Medical Center, NC). Species identity checks on these murine cell lines were performed murine-specific MHC antibodies, and were tested for contamination within the past 6 months using a Mycoplasma Detection Kit (SouthernBiotech, Birmingham, Alabama). 6–8 week old C3H mice were obtained from Charles River Laboratories (Wilmington, MA) for use in SCCVII tumor treatment models. All mice were monitored daily and tumor measurements taken every 2–3 days by staff trained in animal care and use. For survival studies mice were euthanized on the day that their tumor exceeded 12mm in any diameter. No mice died before this endpoint was reached. Groups consisted of 6–8 mice per experimental group and all were euthanized for reaching the humane endpoint, except those mice cured of tumor that were euthanized at approximately 60 days following tumor challenge. Mice in these studies exhibited no overt symptoms through tumor progression or treatment and required no special treatments or housing.

### Cytokine response

To assess the cytokine response of papilloma to STING ligand, papilloma were removed from mice, dissected under sterile conditions into 1-2mm fragments and these were placed in complete media in the presence or absence of 25μg/ml CDN. The papilloma explants were incubated overnight at 37°C then supernatants were collected for multiplex cytokine analysis. Cytokine levels in the supernatants were detected murine multiplex bead assays (Life Technologies, Grand Island, NY) and read on a Luminex 100 array reader. Cytokine concentrations for replicates of each sample were calculated according to a standard curve.

### Immunohistology

Archived tissue blocks were obtained and 5μm sections were cut and mounted for analysis. Tissue sections were boiled in EDTA buffer for antigen retrieval. Sections were first stained with rabbit anti-STING (Cell Signaling Technologies, Danvers, MA) and primary antibody binding was detected with HRP conjugated secondary antibodies followed by DAB development and counterstaining. Images were acquired using a Leica SCN400 whole slide scanner. For immunofluorescence staining, sections were stained with anti-CD3 (Spring Bio, Pleasanton, CA) and primary antibody binding was visualized with AlexaFluor 568 conjugated secondary antibodies (Molecular Probes, Eugene, OR) and mounted with DAPI (Invitrogen, Carlsbad, CA) to stain nuclear material. Images were acquired using a Zeiss Axio observer Z1 with attached Nuance Multispectral Image camera and software (Perkin Elmer, Wlatham, MA). All images displayed in the manuscript are representative of the entire section and their respective experimental cohort.

### Patient selection

Representative blocks of archived tissue from 5 patients for each histology were obtained and sectioned. In order to represent distinct types of oral dysplasia, we identified examples of benign dysplasia, inflammatory conditions including lichen planus and candida ulcer, as well as examples of mild, moderate and severe dysplasia. To represent HPV-asssociated dysplasia and premalignancy we identified examples of benign dysplasia, chondyloma, anal intraepithelial neoplasia grade 3 (AIN3), and cervical intraepithelial neoplasia grade 3 (CIN3). Tissues were sectioned and stained for STING as above. The degree of STING staining was scored by the reviewing pathologist.

### HPV^+^ and HPV- HNSCC array information

A panel of paraffin embedded tissue blocks was used to generate an array of tumors of mixed HNSCC origins using a Perkin Elmer tissue microarrayer. Patient tumors were classified as HPV^+^ if they scored as positive for p16 and originated in the oropharynx. Samples that were negative for p16 and/or originated outside the oropharynx were scored as HPV^-^. Arrayed tissues were sectioned and stained for STING as above. The STING staining score was determined by automated image analysis of tumors, grading the number of STING positive cancer cells and their staining intensity to generate an expression score.

### SCCVII expressing STING

SCCVII were transfected with a plasmid vector expressing murine STING (pUNO1-mSTINGwt, Invivogen) and grown under antibiotic selection to generate stable clones expressing STING. To confirm expression, cells were lysed in RIPA buffer and denatured in SDS loading buffer containing β2-mercaptoethanol, electrophoresed on 10% SDS-PAGE gels and transferred to nitrocellulose. Blocked blots were probed overnight at 4°C with primary antibodies followed by HRP-conjugated secondary antibodies. Binding was detected using a Pierce SuperSignal Pico Chemiluminescent Substrate (Thermo Fisher Scientific, Rockford, IL) and exposure to film. To confirm response to STING ligands, cells were left untreated, treated with 25μg CDN or with 10ng/ml IFNγ. 24hr later cells were analyzed by flow cytometry for expression of MHCI (H2K_k_).

#### Statistics

Data were analyzed and graphed using Prism (GraphPad Software, La Jolla, CA). Individual data sets were compared using Student’s t-test and analysis across multiple groups was performed using ANOVA with individual groups assessed using Tukey’s comparison.

## Results

Pdx-Cre^+/-^ Kras^(G12D)+/-^ Trp53^(R172H)+/-^ mice were observed to develop papilloma at variable penetrance, as has been previously reported [4, 5]. We found no association between papilloma formation and progression of pancreatic adenocarcinoma in the mice, and generally papillomas were present before progression to invasive carcinoma in the pancreas. Some mice developed more than one papilloma, with the location restricted to the periauricular and anogenital regions. As reported in the literature, papilloma formation required both PDX-Cre and Kras^(G12D)^ genotypes suggesting a genetic origin [5]. Prior literature failed to find an infectious origin for these papilloma [5], and in our colony, despite co-housing, papilloma were never found in PDX-Cre^-^ animals nor PDX-Cre^+^ Trp53^(R172H)+/-^ mice that lacked Kras^(G12D)^. Mice were unperturbed by the papilloma, and though individual papilloma could attain large size, we found no evidence of progression to invasive carcinoma, although tandem progression of pancreatic adenocarcinoma in these mice precluded long-term follow up of the papilloma, *per se*. Histological analysis of the papilloma demonstrated significant thickening of the skin with formation of classical keratinizing papilloma as reported [5].

**Fig 1:**
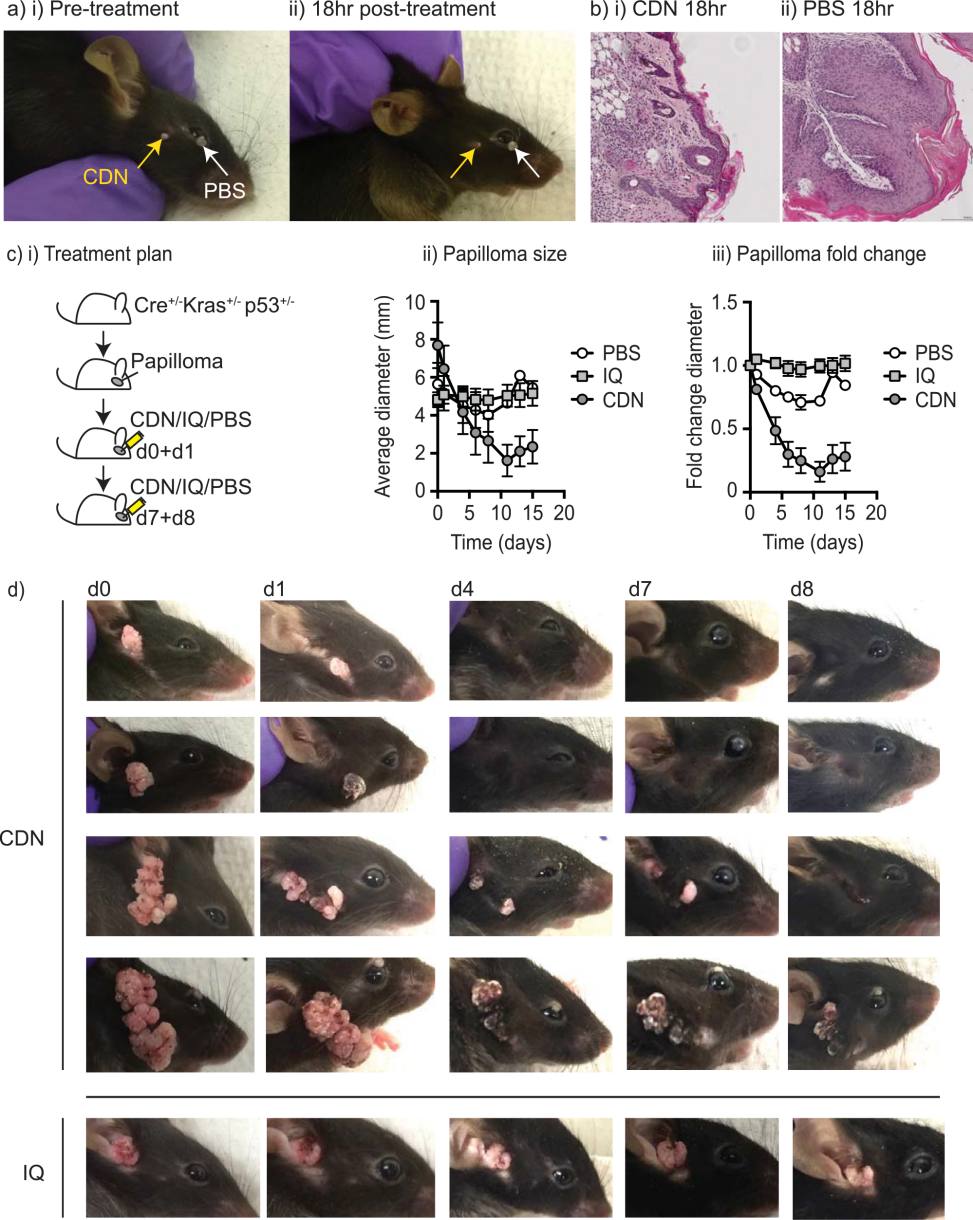
Treatment of murine papilloma with STING ligand. a) A Pdx-Cre^+/-^ Kras^(G12D)+/-^ Trp53^(R172H)+/-^ mouse exhibiting dual small papilloma was i) injected with 25μg CDN in 5μl PBS to one lesion, and PBS alone to the other. ii) 18 hour later the CDN-injected lesion demonstrated regression. b) Papilloma treated with i) CDN or ii) PBS were harvested and examined by histology. c) i) Groups of Pdx-Cre^+/-^ Kras^(G12D)+/-^ Trp53^(R172H)+/-^ mice with large papilloma were randomized to receive treatment with 25μg CDN (n = 9), 25μg Imiquimod (n = 9), or PBS vehicle (n = 3) injected on d0, 1, 7 and 8. ii) average size of papilloma through treatment. iii) Fold change in papilloma size through treatment. d) Representative images of mice bearing papilloma treated as in c) imaged on d0, 1, 4, 7 and 8. Graphs show mean and standard error of each measurement.

To determine whether the papilloma could be treated using STING ligands, mice bearing papilloma were randomly assigned to treatment with a STING activating cyclic dinucleotide (CDN), or PBS vehicle control. In an example of a mouse with two small papillomas on the face, one was treated by direct injection of CDN, and the other given vehicle control (**Fig 1Ai**). These small CDN-injected papilloma rapidly reverted to skin with normal appearance following treatment (**Fig 1Aii**). The site exhibited slight reddening, and histological analysis demonstrated an inflammatory infiltrate in the subcutaneous space (**Fig 1B**). Interestingly, injection of STING ligand into histologically normal skin at distant sites or in normal mice had no observable effect (not shown). Thus, CDN injection appears non-toxic at these doses and causes rapid, site-specific regression of experimental papilloma.

We then tested treatment of larger, 3-15mm papilloma with STING ligands. A single injection of 25μg CDN to each papilloma resulted in rapid loss of papilloma around the injection site, but not full regression. Therefore, we developed a treatment course consisting of injections on d0 and d1, and again on d7 and d8, as the papilloma decreased in size (**Fig 1Ci**). Mice were randomized to receive CDN, conventional treatment with the TLR7 ligand Imiquimod or PBS vehicle. While Imiquimod did not significantly affect the size of the papilloma, CDN treatment resulted in significantly reduced papilloma size (**Fig 1Cii-iii**) (p<0.001 days 4–15). The CDN-treated papilloma showed evidence of blackening (**Fig 1D**), which was suggestive of the hemorrhagic necrosis we have previously observed in STING ligand treatment of advanced cancers [6]. Treatment resulted in complete regression of some papilloma, with skin returning to normal appearance without skin breaks and with the return of hair. Histological examination of CDN-treated papilloma demonstrated regions of inflammatory infiltrate, but no major areas of necrosis (not shown). These data demonstrate that in this mouse model of papilloma, CDN is superior to Imiquimod and results in rapid local regression of papilloma.

**Fig 2:**
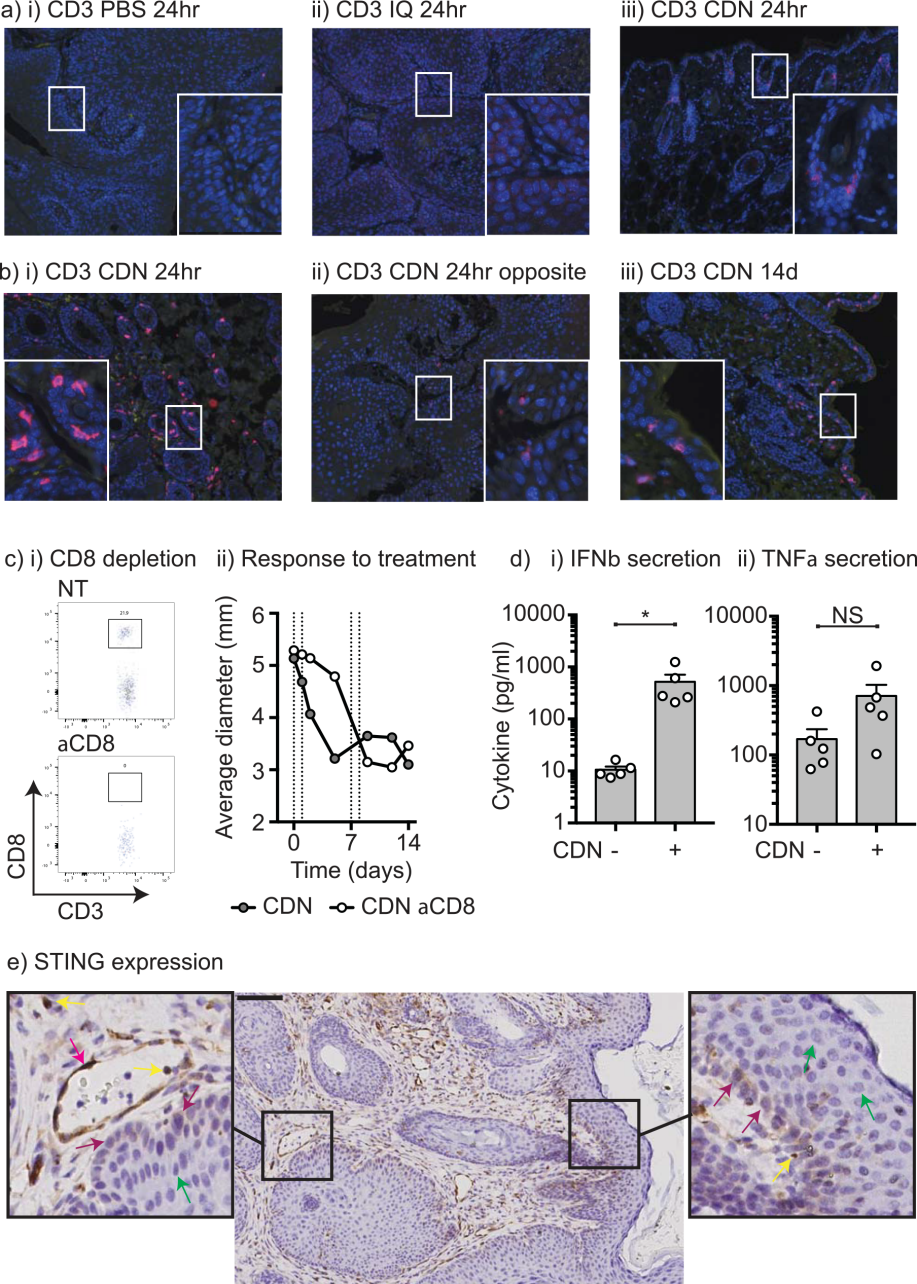
Infiltration of T cells following treatment with STING ligand. Pdx-Cre^+/-^ Kras^(G12D)+/-^ Trp53^(R172H)+/-^ mice exhibiting papilloma were injected with i) PBS vehicle, ii) 25μg Imiquimod, or iii) 25μg CDN and the site harvested 24 hours later for histology. Sections were stained for CD3 (pink) and DAPI nuclear counterstain (blue). Insets are an enlargement of regions of interest. b) A Pdx-Cre^+/-^ Kras^(G12D)+/-^ Trp53^(R172H)+/-^ mouse exhibiting dual papilloma on opposite sides of the face was injected with 25μg CDN to one lesion and the other left untreated. 24 hours later both papilloma sites were harvested and stained for CD3 (pink) and DAPI nuclear counterstain (blue) on the i) treated and ii) untreated opposite side papilloma. iii) CD3 (pink) and DAPI nuclear counterstain (blue) in a CDN-treated papilloma 14 days following initiation of treatment. Insets are an enlargement of regions of interest. c) Pdx-Cre^+/-^ Kras^(G12D)+/-^ Trp53^(R172H)+/-^ mice exhibiting papilloma were left untreated or depleted of CD8 T cells with 50μg anti-CD8 ip. 1 day prior to treatment with 25μg CDN injected on d0, 1, 7 and 8. Detection of CD3^+^CD8^+^ T cells in the peripheral blood on d0 in representative i) NT or ii) CD8-depleted mice. iii) Average size of papilloma through treatment. d) Explants of papilloma were left untreated or treated with 25μg/ml CDN overnight, and supernatants were assessed for section of i) IFNβ and ii) TNFα by multiplex bead assay. e) Immunohistology for STING expression in Pdx-Cre^+/-^ Kras^(G12D)+/-^ Trp53^(R172H)+/-^ murine papilloma. Insets are an enlargement of regions of interest. Arrows depict STING^+^ endothelial cells (red arrows), STING^+^ immune cells (yellow arrows), STING^+^ basal cells (purple arrows), and STING^-^ differentiated non-basal epithelial cells (green arrows).

To investigate whether the response involved adaptive immunity, papilloma were stained for infiltrating CD3^+^ T cells. Few to no T cells were detected in PBS or Imiquimod-treated papilloma (**Fig 2Ai-ii**), but 24 hours following CDN treatment CD3^+^ T cells were found associated with the epidermis in the treatment site (**Fig 2Aiii**). In examples where mice exhibited more than one papilloma, CDN treatment resulted in T cell infiltration in the treated papilloma with poor infiltration to untreated sites (**Fig 2Bi-ii**). This is consistent with the observed site-specific response to CDN injection (**Fig 1**). T cells remained enriched in the epidermis of the treatment site 14 days following treatment (**Fig 2Biii)**. To determine whether T cells were required for papilloma control by STING ligands, mice were depleted of CD8 T cells 1 day prior to initiation of STING therapy (**Fig 2Ci-ii**). Control of papilloma by CDN treatment occurred regardless of the presence of CD8^+^ T cells (**Fig 2Ciii**), consistent with an innate rather than an adaptive immune control of disease. To measure innate cytokines that were released following treatment with STING ligands, explants of papilloma were treated with STING ligands and cytokine secretion was measured by multiplex bead assay. Treatment with STING ligands resulted in a significant secretion of IFNβ, and a trend towards increased TNFα secretion, but this did not reach statistical significance (**Fig 2D**) suggesting that as with other innate stimuli used to treat papilloma, production of type I IFN may be a major mechanism of action. To further understand the mechanism of innate rejection by CDN, we examined the cell populations that express STING in the papilloma. We know from our prior studies that STING is not consistently expressed by all cell types; for example, pancreatic acinar cells do not express STING, but the normal ductal cells of the pancreas and transformed pancreatic ductal adenocarcinoma cells express STING [6], and STING is widely expressed by immune cell populations (reviewed in [9]). Histological staining of STING in the murine papilloma demonstrated that the basal cell layer of the papilloma expresses STING but this is lost on differentiation to keratinocyte layers (**Fig 2E**). However, endothelial cells and infiltrating immune cells underlying the papilloma strongly express STING (**Fig 2E**), and these cells are also the likely target for STING ligands [6, 7].

**Fig 3:**
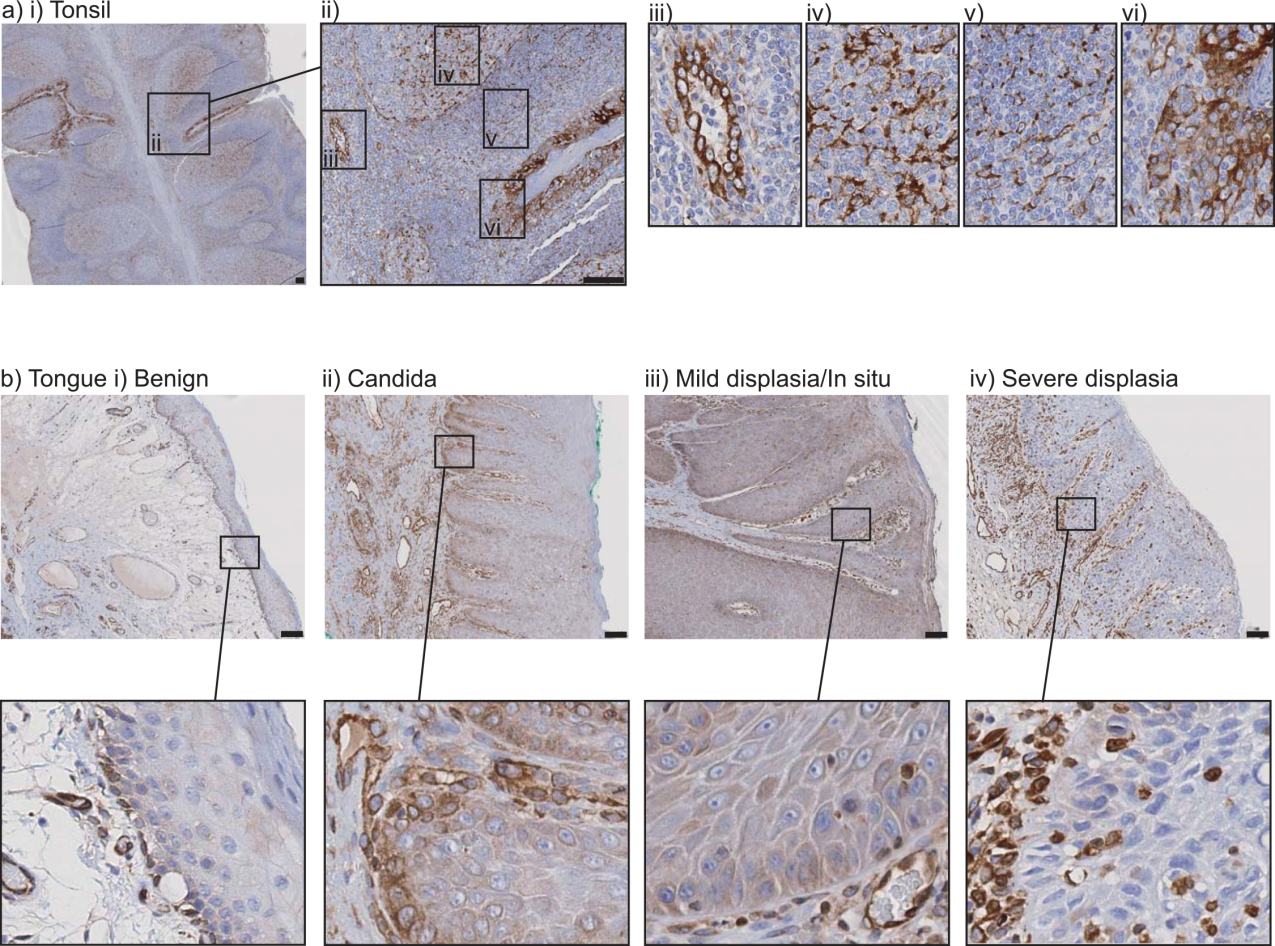
STING expression in oral dysplasia. a) Staining for STING in normal tonsil. Highlighted areas include positive iii) endothelia, iv) follicular dendritic cells, v) interdigitating cells, vi) tonsilar crypt. b) Staining for STING in i) benign dysplasia, ii) candida infection, iii) mild dysplasia/in situ, or iv) severe dysplasia.

To understand whether this expression pattern is shared in patients, we examined STING expression in normal human tonsil and in a panel of human benign and premalignant oral dysplasia. Representative examples are shown in **Fig 3**. Immunohistology demonstrated strong expression of STING in cells of the tonsil (**Fig 3Ai-ii**), in particular, strong staining was observed in the high endothelial venules (**Fig 3Aiii**), in cells within the light zone of the germinal center that are consistent with follicular dendritic cells (**Fig 3Aiv**), and in cells within the T cell zone that are consistent with interdigitating cells (**Fig 3Av**). STING expression was readily detectible in immune cells underlying normal tonsillar epithelia, but was particularly high in select tonsillar crypts **(Fig 3Ai and vi**), suggestive of ongoing local responses to bacterial or viral exposure. In normal tongue, STING expression was restricted to the basal cell layer and lost upon differentiation to keratinocyte layers (**Fig 3Bi**). In some areas of oral candidiasis associated with benign thickening, STING expression was enhanced (**Fig 3Bii**). In mild or severe dysplasia of the oral tongue, STING expression was mixed but did not increase with the degree of dysplasia (**Fig 3Biii-iv**), and occasionally STING expression was not detected in the squamous cells of severe oral dysplasia (**Fig 3Biv**). However, in each case, expression of STING was readily detectable in endothelial cells and in immune cells in the submucosal layer underlying the squamous layer. These data show that STING is expressed by basal cells in the oral mucosa, which are a key target for HPV infection, but there is no significant association with STING expression and the progression of dysplasia in the tongue or oral cavity.

**Fig 4:**
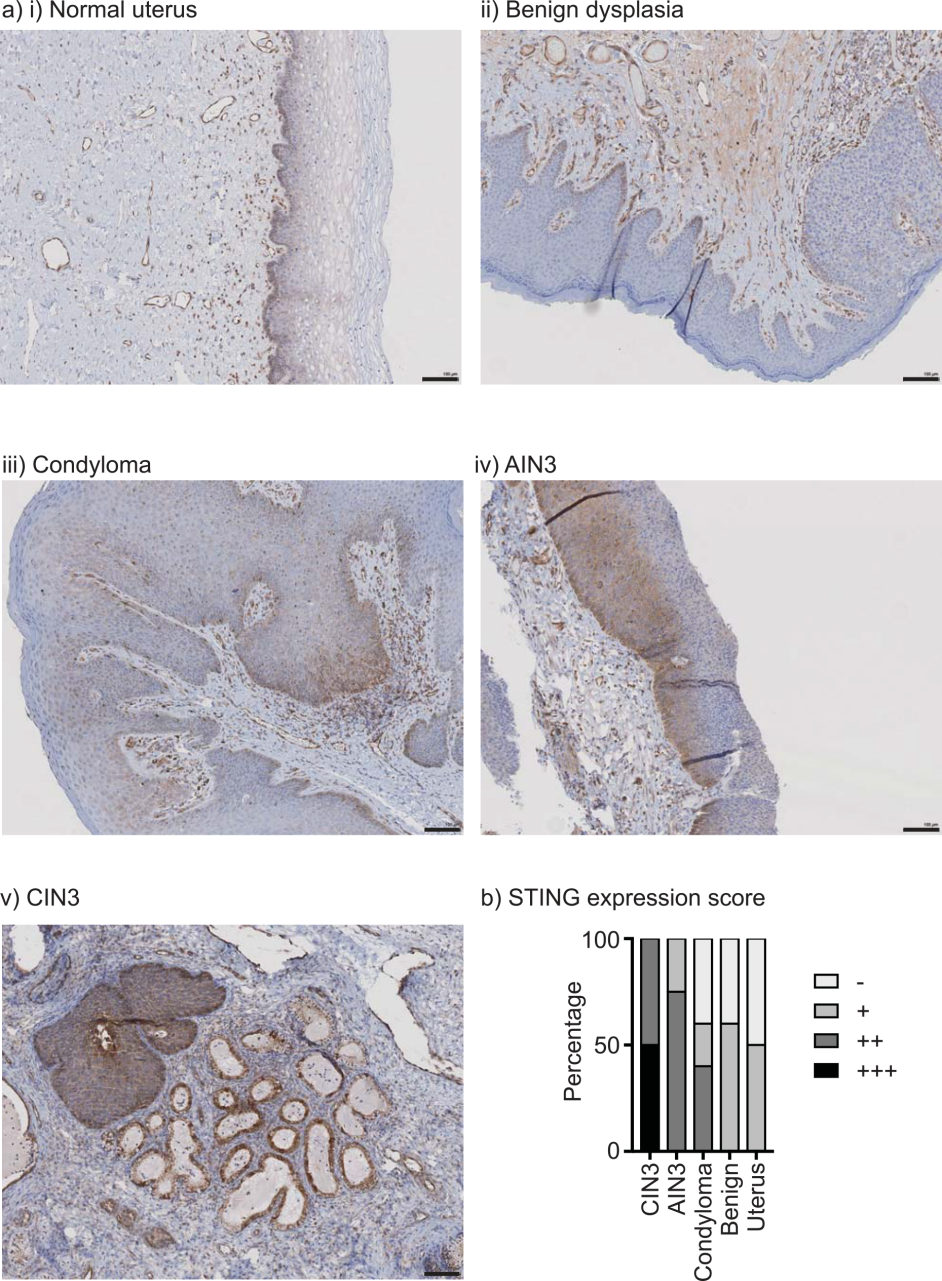
STING expression in HPV-associated disease. a) Examples of STING staining in i) normal uterus, ii) benign dysplasia, iii) condyloma, iv) AIN3, v) CIN3. b) The degree of STING staining was scored on a scale from negative (-) to highly positive (+++). The graph shows a summary of the proportion of each histology with each staining pattern.

While HPV is known to result in HPV-associated cancers in the palatine and lingual tonsils, premalignant HPV^+^ neoplasia are rarely observed in either site. To evaluate STING expression in HPV-associated premalignancies, we performed immunohistology of high grade cervical intraepithelial neoplasia (CIN3), high grade anal intraepithelial neoplasia (AIN3), and condyloma, as well as benign hyperplasia and tissues from normal uterus as a control. The pattern of normal tissue STING expression was very similar to that seen in the tonsil and tongue, with predominant expression in the basal cells and underlying immune cells, and loss upon differentiation into the keratinocyte layer (**Fig 4A**). STING was also expressed in all HPV-associated dysplasia, with a trend towards increased expression of STING in CIN3 (**Fig 4A and 4B**). In addition, in all cases, STING was expressed in endothelial structures and immune cells underlying the squamous epithelium. These data demonstrate that STING is expressed in basal cells and expression is maintained or increased in these cells through HPV^+^ dysplastic progression.

**Fig 5:**
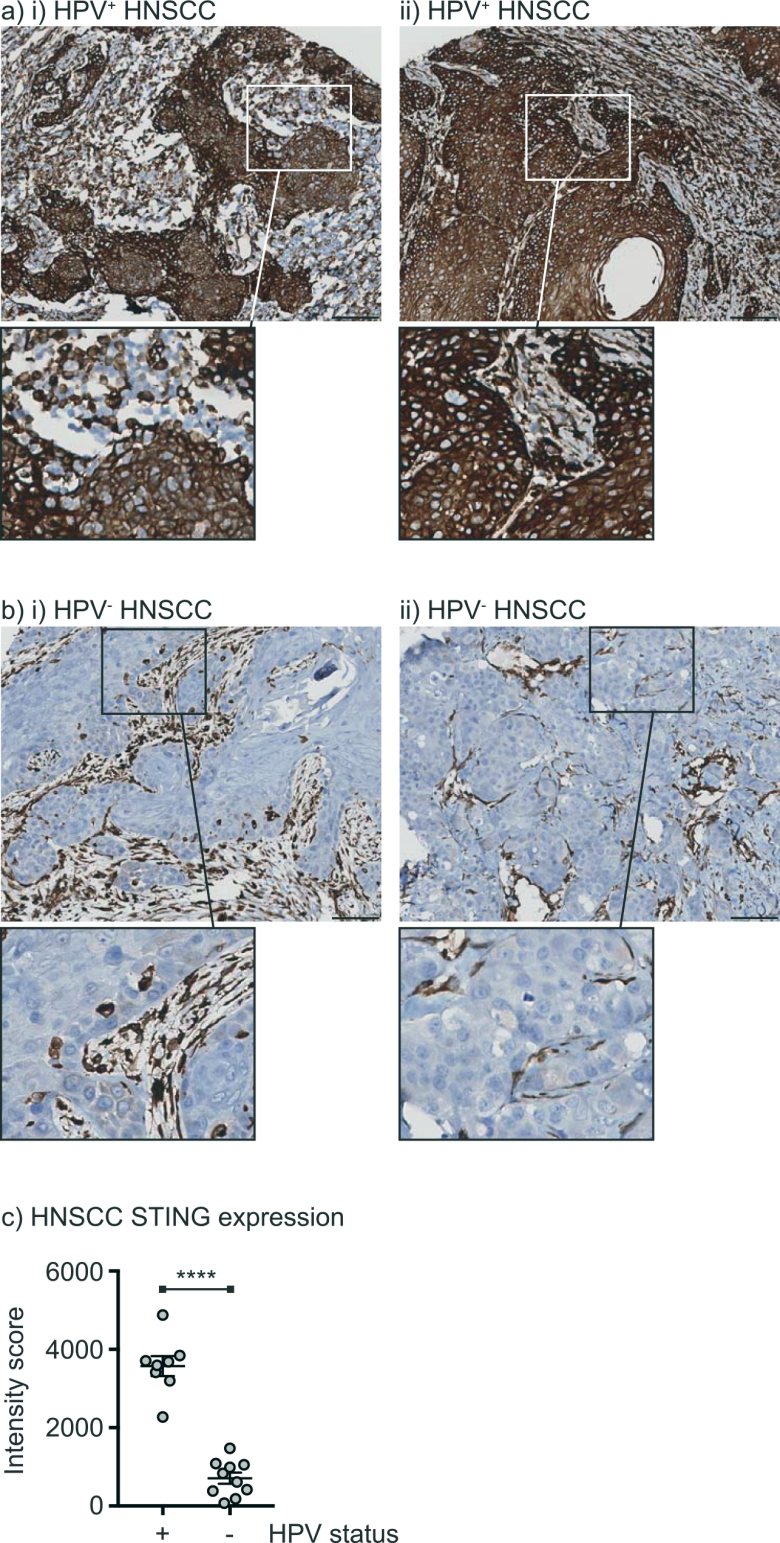
STING expression in HPV^+^ and HPV^-^ HNSCC. a) Examples of STING staining in two examples of HPV^+^ HNSCC. b) Examples of STING staining in two examples of HPV^-^ HNSCC. c) Intensity of STING expression in HPV^+^ and HPV^-^ HNSCC. Each symbol represents one patient. **** = p<0.0001.

To examine STING expression in advanced HPV^+^ and HPV^-^ cancers, we stained a tissue array from a panel of HNSCC from various anatomic sites. The tumors were classified as HPV^+^ if they scored as positive for p16 by immunohistochemistry and originated in the oropharynx. Samples that were negative for p16 and/or originated outside the oropharynx were scored as HPV^-^. In all cases, STING was detectable in immune cells in the vicinity of the tumor. However, HPV^+^ HNSCC exhibited high levels of STING expression in the cancer cells (**Fig 5Ai-ii**), while HPV^-^ HNSCC cancer cells exhibited low or absent STING expression (**Fig 5B i-ii**). This expression was quantified using image analysis software according to the proportion of cancer cells with high STING staining in the cytoplasm, confirming that HPV^+^ HNSCC exhibited significantly higher STING expression in cancer cells than HPV^-^ HNSCC (**Fig 5C**). These data suggest that HPV^+^ cancer cells preserve STING expression through malignant progression from their basal cell origin, in contrast to HPV^-^ HNSCC, which is generally composed of keratinizing, non-basaloid epithelium that poorly express STING. This is consistent with the loss of STING expression during normal squamous differentiation.

**Fig 6:**
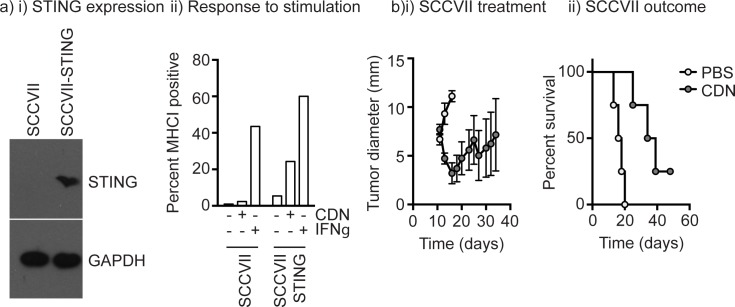
Response to STING ligands in STING^-^ murine HNSCC. a) i) SCCVII cells or SCCVII cells stably expressing STING (SCCVII-STING) were lysed and western blotted for STING expression and GAPDH as a loading control. ii) SCCVII cells or SCCVII-STING in culture were left untreated or treated with 25μg/ml CDN or 10ng/ml IFNγ for 24 hours then analyzed by flow cytometry for MHCI expression. b) Parental, unmodified SCCVII tumors in C3H mice were treated by intratumoral injection of PBS (open symbols) or 25μg CDN in PBS (filled symbols) on d10 and d11 following tumor implantation. i) Graphs show average tumor diameters and ii) overall survival.

These data would appear to suggest that therapies targeting STING would be ineffective in HPV^-^ HNSCC; however, host rather than cancer cell expression of STING has been shown to be critical for therapies targeting STING [6, 10]. To test therapies in the absence of STING expression in cancer cells, we made use of the murine SCCVII squamous cell carcinoma line commonly used to model HNSCC in immune competent mice [11]. We identified that this line lacks STING expression (**Fig 6Ai**) in contrast to our prior work with the Panc02 pancreatic adenocarcinoma cell line that expresses STING and can directly respond to STING ligands *in vitro* [6]. To confirm lack of functional STING expression, SCCVII cells were treated with CDN, which results in IFN-mediated upregulation of MHCI in responsive cells. SCCVII cells cannot upregulate MHCI following direct treatment with CDN, but readily upregulate MHC in response to IFNγ treatment (**Fig 6Aii**). Stable expression of STING in these cells restores responses to direct treatment with STING ligands (**Fig 6Ai-ii**), confirming that unmodified SCCVII cells cannot directly respond to STING ligands. Despite increases in MHC following treatment with CDN, secretion of type I IFN was below the detection level of our assay, suggesting that the engineered cancer cells are weakly responsive to in vitro stimulation. To evaluate whether STING unresponsive SCCVII tumors could respond to treatment with STING ligands *in vivo*, unmodified parental SCCVII tumors were established in immunocompetent C3H mice and once established, treated with 2 daily injections of PBS alone or 25μg CDN in PBS. CDN treatment resulted in a rapid, though transient tumor regression, which significantly enhanced survival of these mice (**Fig 6B**). These data demonstrate that STING ligands are effective where cancer cells lack STING expression.

## Discussion

Despite anecdotal efficacy and good results in single arm studies, Imiquimod and type I IFN have not demonstrated efficacy in randomized clinical studies of HPV-associated premalignant disease. There remains a need for alternative agents to treat HPV-associated disease and prevent progression into invasive cancer in high-risk groups. Ligands targeting the STING pathway have been shown to activate potent inflammatory responses *in vitro* and *in vivo*, and can result in dramatic regression of tumors in mice. We demonstrate in a mouse papilloma model that the STING ligands are superior to Imiquimod for regression of papilloma, yet is non-toxic to normal skin. We demonstrate that STING is expressed in the basal layer of normal tonsil and in tonsillar crypts, which are a presumed site of HPV infection. STING expression is preserved in HPV-associated premalignancies and in HPV^+^ HNSCC, but lacks association with non-HPV oral dysplasia and HNSCC. Nevertheless, via effects on host cells STING ligands can treat tumors derived from cancer cells that lack STING expression.

We acknowledge limitations inherent to the Pdx-Cre^+/-^ Kras^(G12D)+/-^ Trp53^(R172H)+/-^ model of papilloma, in particular the ongoing progression of tandem pancreatic tumors. However, the genetic development of papilloma in these mice is similar to other squamous cell carcinoma murine models. A number of systems are based on the K14 promoter to ensure activation of oncogenes in basal keratinocytes. Mice with K14 driven expression of Cre bred with the same Kras^(G12D)fl/fl^ floxed mice used in our study, develop variably situated benign papilloma and additional cross with the same Trp53^(R172H)fl/fl^ mice used in our study resulted in variably situated invasive carcinoma [12]. Thus, it is possible that the papilloma in our Pdx-Cre^+/-^ Kras^(G12D)+/-^ Trp53^(R172H)+/-^ mice have the potential to become invasive carcinoma were it not for the dominant progression of the lethal pancreatic tumors. Mutation of Ras family members and mutation or loss of heterozygosity of *Trp53* are commonly detected in models of mutagen-driven squamous cell carcinoma [13], and the mutational profile was shown to be very similar to that of human squamous cell carcinoma [13]. K14-driven expression of HPV16 E6 and E7 results in a systemic epidermal dysplasia and low penetrance oncogenesis [14]; however, by combining this with the 4-NQO model of chemical carcinogenesis, which induces high penetrance formation of multifocal papilloma that can progress to malignancy [15], the rate of progression can be significantly advanced within the oral cavity (and esophagus) [16]. Similarly, cervical oncogenesis can be driven in mice with K14-driven HPV16 E6 and E7 oncogenes via chronic local estrogen administration [17, 18]. Models such as these would be ideal to further study STING ligands as therapeutics that can treat papilloma *in vivo* and measure their ability to prevent the development of invasive carcinomas in immune competent animals. However, in view of our data that STING is expressed in the basal cell layer of the papilloma, but that this is lost on differentiation to keratinocyte layers, this may explain why HPV positive tumors express STING whereas HPV negative tumors do not. HPV is known to infect the basal layer of stratified epithelium whereas non-HPV squamous cell carcinoma tends to originate from the more differentiated keratinizing layers of the epithelium. We propose that STING expression is linked to the basaloid squamous cell origin of HPV^+^ cancer, and is not a result of HPV infection or due to expression of HPV-associated oncogenes. Therefore, the presence or absence of STING expression in these alternative models will be determined by the cell origin of oncogenesis rather than the presence of HPV genes.

While we show that STING is not frequently expressed in HPV^-^ HNSCC, this does not necessarily preclude ligands targeting STING as a therapeutic approach in HPV^-^ HNSCC. We have previously shown that STING expression in the host and not the cancer cell is critical for responses to STING ligands and radiation therapy [6] and others have shown that host STING is critical for responses to STING ligands alone [10]. As we demonstrate here using a STING^-^ HNSCC model, STING expression by the cancer cell is not essential for the response to STING ligands. We see similar responses to STING ligands in tumors where the cancer cells express STING as in those where the cancer cells do not express STING [6]. In these invasive carcinomas the mechanism of tumor control likely depends on TNFα and type I IFN production by host cells, in particular via myeloid cells in the tumor environment, which in turn initiate and propagate adaptive anti-tumor immune responses (6, 7). Recent data suggests that cancer cell expression of STING is critical for the full effect of radiation therapy (19). While this is not clear evidence for the therapeutic responsiveness of HPV positive head and neck cancer, it is one possible explanation for the difference in response that is seen clinically. In the papilloma model it is probable that the oncogene-driven benign papilloma lack the mutated neoantigenic targets that would be necessary for an effective T cell-mediated adaptive immune response [13]. Adaptive immune responses take time to develop and would be unlikely to explain the rapid responses observed in the papilloma following administration of CDN. We demonstrated that CDN were equally able to cause papilloma regression in the absence of CD8 T cells. Therefore, the rapid and sustained presence of T cells we observed in the CDN-treated papilloma are likely due to treatment-induced inflammation, rather than mediating papilloma regression, *per se*, and is consistent our data showing induction of type I IFN in the papilloma. We previously demonstrated that early control of invasive carcinoma treated with STING ligand was dependent on innate cytokines and was preserved in CD8-depleted mice as well as Rag^-/-^ mice lacking T cells [6]. However, the adaptive immune response, mediated by CD8 T cells, was required for long term control in these experiments by preventing tumor recurrence [6]. Therefore, the role of T cells may vary according to the timeline of response. Basal cell carcinoma can respond to direct treatment with innate cytokines [19], which can directly cause pro-apoptotic and anti-proliferative effects on the hyperplastic basal cell layer [20, 21]. If innate cytokines are the dominant mechanism of action, for example in Imiquimod mediated rejection of actinic keratosis in immunosuppressed transplant recipients, adaptive immunity may be unnecessary [22]. In the histology tissue sections we examined, STING was expressed in immune cells in close contact with the basal cells and in close contact with both HPV^+^ and HPV^-^ tumors. We and others have shown that myeloid cells respond strongly to STING ligands [6, 7], and these, rather than the dysplastic epithelial or cancer cells may be the target cells for STING ligands administered *in vivo*. Thus, treatment with STING ligands may depend on host cells producing TNFα and type I IFN, and these cytokines then act as effectors. However, we cannot at present exclude direct recognition of STING ligands by STING expressed in abnormal basal cells as the cause of papilloma regression in our model. Further studies are necessary to evaluate the mechanisms of papilloma control, the relative role of innate cytokines, adaptive immunity and the relative response rates in STING^+^ and STING^-^ cancers.

While our major goals in developing this therapy are preventing progression of premalignant disease to lethal invasive cancer and treatment of invasive cancer, non-progressing benign HPV-associated papilloma remain a significant public health concern. The non-ablative rapid regression of papilloma following application of STING ligands could be highly applicable to condyloma, verrucae, or warts. Moreover, since this therapy was effective without onboard viral-associated genes, this therapy could be applicable to intraductal papilloma and choroid plexus papilloma that are of unknown cause. We propose that STING represents a novel innate immune target for treatment of benign dysplasia and premalignancy involving basal cells, in addition to its emerging role as a therapy for invasive carcinomas.
