# Correction: STING expression and response to treatment with STING ligands in premalignant and malignant disease

**DOI:** 10.1371/journal.pone.0192988

**Published:** 2018-02-15

**Authors:** Jason R. Baird, Zipei Feng, Hong D. Xiao, David Friedman, Ben Cottam, Bernard A. Fox, Gwen Kramer, Rom S. Leidner, R. Bryan Bell, Kristina H. Young, Marka R. Crittenden, Michael J. Gough

The second author’s name is spelled incorrectly. The correct name is: Zipei Feng.
